# Healthy lifestyle choices and microvascular complications: New insights into diabetes management

**DOI:** 10.1371/journal.pmed.1004152

**Published:** 2023-01-10

**Authors:** Yogini V. Chudasama, Kamlesh Khunti

**Affiliations:** Leicester Real World Evidence Unit, Diabetes Research Centre, Leicester General Hospital, University of Leicester, Leicester, United Kingdom

## Abstract

Yogini Chudasama and Kamlesh Khunti discuss new evidence, published in PLOS Medicine, highlighting the importance of healthy lifestyle behaviours in diabetes management.

Diabetes is a progressive disease and, if not adequately managed, can lead to a number of health complications including diabetes-related macro or microvascular diseases and further clustering of comorbidities [1,2]. While diabetes medications and treatments contribute substantially to managing risk factors, relying on medication alone will not help solve the long-term complications of diabetes [3]. It is evident that healthy lifestyle behaviours are equally as important [3], yet many individuals have an unhealthy lifestyle, often based on interpersonal influences, economic or social pressures [4]. Many studies have investigated the association of lifestyle factors with, for example, risk factor control, cardiovascular risk, or mortality [5]. To date though, there is a lack of evidence reporting on the association of lifestyle factors with the risk of microvascular complications. Understanding the benefits of healthy lifestyle behaviours at early stages of diabetes would potentially result in fewer patients living with severe outcomes developing multiple long-term conditions, ultimately minimising the impact on the healthcare system [6].

In this issue of *PLOS Medicine*, an accompanying study by Tingting Geng and colleagues provides a new perspective on the association of overall lifestyle factors with the risk of microvascular complications [7]. This retrospective study included 15,104 people with type 2 diabetes free of macro or microvascular complications at baseline from the UK Biobank. The healthy lifestyle behaviours that formed the study exposure included not smoking currently, recommended waist circumference level, regular physical activity, a healthy diet, and moderate alcohol drinking. Study outcomes were diagnosis of diabetic retinopathy, diabetic neuropathy, and diabetic kidney disease, determined via data linkage with hospital inpatient admissions and death registries. Over a median follow-up of 8.1 years, being more adherent to healthy lifestyle behaviours (adhering to 4 to 5 low-risk lifestyle behaviours versus 0 to 1) was associated with a significantly lower risk of diabetic retinopathy [hazard ratio 0.65 (95% confidence interval 0.46, 0.91)], diabetic kidney disease [0.43 (0.30, 0.61)], and diabetic neuropathy [0.46 (0.29, 0.74)], as well as the composite of microvascular complications [0.54 (0.43, 0.68)]. Secondly, the population attributable fraction (PAF) of diabetic microvascular complications ranged from 20.3% to 39.0% for lifestyle factors, and biomarkers albumin, HDL-C, apolipoprotein A, triglycerides, C-reactive protein, and HbA1c collectively explained 27.2 (15.1, 44.0)% of the associations with microvascular complications. The new findings contribute to the evidence on healthy lifestyle modifications, with the rich data and comprehensive sensitivity analyses as the main strengths of this study.

A similar recent study, involving 26,004 patients with diabetes in the China Kadoorie Biobank with a median follow-up of 10.2 years, also found that combined low-risk lifestyle factors were associated with a lower risk of microvascular complications [0.75 (0.62 to 0.91)], especially diabetic nephropathy [8]. Thus, the 2 observational studies taken from different study populations give us confidence in the positive findings of lifestyle interventions in the prevention of microvascular complications. However, both highlight that there is an extremely low number of people with diabetes who adhere to a healthy lifestyle: in the new study, Tingting Geng and colleagues found only 10.3% were adherent to 4 to 5 low-risk lifestyle behaviours in UK Biobank [7]. Similarly, Zhijia Sun and colleagues found only 14.4% were adherent in the Chinese Kadoorie Biobank [8]. Together, these studies further emphasise the importance of establishing an overall healthier lifestyle at the earlier stages of diabetes and promoting population and policy-level interventions [9].

Populations across many countries globally are increasingly ethnically diverse [10]. For instance, the population of the United Kingdom includes large populations born in Poland, India, Pakistan, the Republic of Ireland, and Germany [10]. A range of cultural aspects that influence lifestyle behaviours therefore have the potential to contribute to diabetes cases and complications [11] and require careful consideration by the research community. Since there has recently been greater attention paid to ethnic and social health inequalities [12,13], further research on lifestyle behaviours and diabetes complications may benefit from stratified analyses to identify whether the findings differ between patient demographics. This may necessitate studies in more diverse cohorts, however, as the UK Biobank data used in the present study is limited in terms of ethnic diversity, with the majority of the population from white ethnicity [7]. Additionally, lifestyle factors in the study by Geng and colleagues were assessed at a single time point [7]; future longitudinal studies need to include measurement of lifestyle behaviours at multiple time points to allow for a flexible modelling approach that accounts for time-varying covariance. This will provide insight into both the extent to which patients’ lifestyle habits change over time and how this affects the incidence and progress of diabetic complications.

While the new paper suggests adhering to an overall healthy lifestyle to lower the risk of diabetic microvascular complications, public health policies ought to also consider targeting key risk factors where instigating behavioural change may be challenging. Previous evidence highlights that young and middle-aged adults from socioeconomically deprived areas (where engaging in a healthy lifestyle could be more challenging) have the highest number of multiple long-term conditions [14]. Another recent UK Biobank study suggested that, although an overall healthy lifestyle was equally important in people with and without multimorbidity in terms of increasing longevity, some individual lifestyle factors (for example, smoking) had a greater impact on life expectancy [15]. Therefore, public health efforts targeting certain key risk factors may be able to help patients reduce their risk of diabetes-related complications. For instance, some public health policies have focused on increasing access to healthy food options in areas with high levels of socioeconomic deprivation, by providing incentives for supermarkets to open in underserved communities or by implementing programs that make fresh fruits and vegetables more affordable [16]. Additionally, some policies have focused on implementing smoking bans, increasing the age of tobacco sales, or promoting vaping, to encourage individuals to quit smoking and reduce the negative health effects of second-hand smoke [17].

This new research provides us with real-world evidence on the association between healthy lifestyle factors with the risk of microvascular complications. Future research should include stratified analyses by patient sociodemographics such as ethnicity, the use of repeated lifestyle measures, and targeting of specific healthy lifestyle factors. As the prevalence of diabetes continues to increase and more people are ageing with the disease, different priorities and health care needs for diabetes care are emerging, particularly among older patients [18]. Relying on medication alone will not help solve the long-term complications of diabetes; healthier lifestyle choices will also be vital in altering the trajectory of diabetes complications globally.
